# Complete Genome Sequence of *Francisella endociliophora* Strain FSC1006, Isolated from a Laboratory Culture of the Marine Ciliate *Euplotes raikovi*

**DOI:** 10.1128/genomeA.01227-14

**Published:** 2014-11-26

**Authors:** Andreas Sjödin, Caroline Öhrman, Stina Bäckman, Adrian Lärkeryd, Malin Granberg, Eva Lundmark, Edvin Karlsson, Elin Nilsson, Adriana Vallesi, Christian Tellgren-Roth, Per Stenberg, Johanna Thelaus

**Affiliations:** a Division of CBRN Defence and Security, Swedish Defence Research Agency, FOI, Umeå, Sweden; b Department of Chemistry, Computational Life Science Cluster (CLiC), Umeå University, Umeå, Sweden; c Laboratory of Eukaryotic Microbiology and Animal Biology, University of Camerino, Camerino, Macerata, Italy; d Uppsala Genome Center, National Genomics Infrastructure-Science for Life Laboratory, Department of Immunology, Genetics and Pathology, Uppsala University, BMC, Uppsala, Sweden; e Department of Molecular Biology, Umeå University, Umeå, Sweden

## Abstract

A strain of *Francisella endociliophora* was isolated from a laboratory culture of the marine ciliate *Euplotes raikovi*. Here, we report the complete genome sequence of the bacterial strain FSC1006 (*Francisella* Strain Collection, Swedish Defence Research Agency, Umeå, Sweden).

## GENOME ANNOUNCEMENT

In 2011, Schrallhammer and colleagues (1) published a 16S rRNA gene sequence from a bacterium associated with the marine ciliate *Euplotes raikovi* that was phylogenetically positioned within the genus *Francisella* and described as “Candidatus *F. noatunensis* subsp. *endociliophora* subsp. nov.” (1). The genus *Francisella* includes several species with little genetic variation but with diverse environmental niches spanning from marine fish pathogens, potential endosymbionts of protozoa, and human pathogens, *viz*. *Francisella tularensis* subsp. *tularensis*, the causative agent of tularemia included among the tier 1 agents on the United States Select Agents list (2–4). The laboratory culture of *E. raikovi* was used to isolate strain FSC1006 (*Francisella* Strain Collection, Swedish Defence Research Agency, Umeå, Sweden). Briefly, the sample was bead beaten and acid treated (5) before being serially diluted in phosphate-buffered saline (PBS) and spread on Thayer-Martin agar culture plates (6). The culture plates were incubated at room temperature (20°C) for 1 to 2 weeks and monitored for bacterial growth. The strain FSC1006 grows in nutrient broth (NB) and McLeod medium at room temperature (RT) but not at 37°C.

DNA was extracted as previously described by Larsson et al. (7) and sent for sequencing to the Uppsala Genome Center (Uppsala, Sweden). A Pacific Biosciences RSII system (10-kb library, 2-h movie length) generated a total of 144,357 PacBio reads, with an average read length of 3.9 kb, using two single-molecular real-time (SMRT) cells. The SMRT Analysis system version 2.2.0.p3 was used to assemble a draft genome consisting of a single contig. Finally, the contig ends were aligned to determine the joining point of the circular genome. The complete genome consists of 2,015,987 nucleotides and has a mean G+C content of 32.4%. Annotation was performed using the NCBI annotation service.

*F. endociliophora* FSC1006 contains 1,831 predicted protein-coding sequences and 49 predicted noncoding RNAs. The average nucleotide identity (ANI) was calculated by pairwise genome comparisons for the publicly available genomes within *Francisella* clades I and II (8), using the MUMmer and BLAST algorithms with JSpecies version 1.2.1 (9). The similarities between *F. endociliophora* and the clade I and clade II genomes were 78.4 to 77.5% and 78.3 to 77.6%, respectively. Commonly, a threshold of >95 to 96% identity is used to classify genomes as belonging to the same species (10). Multiple genome alignments were computed using progressiveMauve, with default parameters (11), and a phylogenetic tree was generated using the neighbor-joining method. The phylogeny shows that *F. endociliophora* does not belong to any of the two previously known *Francisella* clades and instead forms a new separate branching clade in the *Francisella* genus.

This complete genome sequence is of great importance for the understanding of the environmental diversity of *Francisella* species. A broadening of our understanding is needed in order to further explore the ecology and epidemiology of *Francisella* spp. (12). We argue, based on the phylogeny and low similarity, that this isolate should be classified as a new species, *F. endociliophora*, instead of being a subspecies of *Francisella noatunensis*.

### Nucleotide sequence accession numbers.

This whole-genome shotgun project has been deposited at DDBJ/EMBL/GenBank under the accession no. CP009574. The version described in this paper is the first version, CP009574.1.
